# RE: White blood cell counts and neutrophil to lymphocyte ratio in the diagnosis of testicular cancer: a simple secondary serum tumor marker

**DOI:** 10.1590/S1677-5538.IBJU.2016.0319

**Published:** 2016

**Authors:** Ozgur Haki Yuksel, Ayhan Verit, Aytac Sahin, Ahmet Urkmez, Fatih Uruc, Zafer Demirer, Ali Güragac, Sami Uguz, Ali Ugur Uslu, Murat Zor

**Affiliations:** 1 Department of Urology, Eskisehir Military Hospital, Eskisehir, Turkey; 2 Department of Urology, Tatvan Military Hospital, Bitlis Turkey; 3 Department of Urology, Gülhane Military Medical Faculty School of Medicine, Ankara, Turkey; 4 Department of Internal Medicine, Eskisehir Military Hospital, Eskisehir, Turkey

*To the editor*,

We read the article by Yuksel et al. (1) entitled “White blood cell counts and neutrophil to lymphocyte ratio in the diagnosis of testicular cancer: a simple secondary serum tumor marker” published in your journal. This is a very interesting study which is very well-designed and presented. Yuksel and coworkers has evaluated the white blood cell (WBC) counts and neutrophil to lymphocyte ratio (NLR) as markers of systemic inflammation in the diagnosis of localized testicular cancer as a malignancy with initially low volume. They have shown that (WBC) counts and NLR were statistically significantly higher in patients with testicular cancer compared with the control group. They have defended that both (WBC) counts and NLR can be used as a simple test in the diagnosis of testicular cancer. We respectfully thank the authors for this contribution.

Testicular cancer is the most common cancer in young men. Commonly, most testicular cancers indicate with palpable mass and are malignant in 90–95% of all cases. Standard treatment for testicular cancer is radical orchiectomy (2–4). Preoperative exhaustive evaluation with physical examination, tumour markers, and ultrasonography, can lead to better diagnosis of testicular cancer. AFP, HCG and LDH were defined as tumor markers in testicular cancer (5). Serum tumour markers are prognostic factors and conduce to diagnosis and staging. Serum tumour markers should be detected before, and 5–7 days after, orchiectomy (5). Tumour markers are of value for diagnosis (before orchiectomy) as well as for prognosis (after orchiectomy).

Routine peripheral blood counts may be beneficial in prognosis and diagnosis of many disorders, involving testicular cancer (1, 6–13). NLR is measured by dividing the number of neutrophils by the number of lymphocytes. NLR is used as an inflammatory marker in the inflammatory disorders. Inflammation has an crucial role in the proliferation, angiogenesis, and metastasis of cancer cells and is substantial in the development and progression of the disease (14, 15).

NLR is easily measurable laboratory marker used to appraise systemic inflammation. These marker maybe related with many circumstances such as thyroid function abnormalities, renal and/or hepatic dysfunction, diabetes mellitus, hypertension, chronic obstructive respiratory disease, metabolic syndrome, malignancy, B12 and folic acid deficiency, inflammatory diseases, local and/or systemic infection, smoking, alcohol consumption, anemia, and any use of medication (immunosuppressive agents corticosteroids and non-steroid anti-inflammatory drugs) related to inflammatory status of patients (6–13). In this study only excluded who patients with an evidence of concomitant infection or inflammation. The authors should have mentioned these factors.

In conclusion, we strongly believe the findings obtained from the current study will lead to further studies examining the WBC counts and NLR in the diagnosis of testicular cancer.
